# Systematic Review of Household Transmission of Strep A: A Potential Site for Prevention That Has Eluded Attention

**DOI:** 10.1093/infdis/jiae136

**Published:** 2024-03-13

**Authors:** Stephanie L Enkel, Samuel Barnes, Jessica Daw, Emma Pearson, Hannah M M Thomas, Nina Lansbury, Rosemary Wyber, Andrew M Redmond, Anna P Ralph, Jonathan R Carapetis, Asha C Bowen

**Affiliations:** Wesfarmers Centre of Vaccines and Infectious Diseases, Telethon Kids Institute, Nedlands, Australia; Medical School, University of Western Australia, Crawley, Australia; School of Public Health, University of Queensland, Brisbane, Australia; Wesfarmers Centre of Vaccines and Infectious Diseases, Telethon Kids Institute, Nedlands, Australia; Wesfarmers Centre of Vaccines and Infectious Diseases, Telethon Kids Institute, Nedlands, Australia; Wesfarmers Centre of Vaccines and Infectious Diseases, Telethon Kids Institute, Nedlands, Australia; School of Public Health, University of Queensland, Brisbane, Australia; Wesfarmers Centre of Vaccines and Infectious Diseases, Telethon Kids Institute, Nedlands, Australia; National Centre for Aboriginal and Torres Strait Islander Wellbeing Research, The Australian National University, Canberra, Australia; School of Public Health, University of Queensland, Brisbane, Australia; Department of Infectious Diseases Unit, Metro North Hospital and Health Service, Queensland Health, Brisbane, Australia; Department of Infectious Diseases, Royal Darwin Hospital, Darwin, Australia; Menzies School of Health Research, Charles Darwin University, Darwin, Australia; Wesfarmers Centre of Vaccines and Infectious Diseases, Telethon Kids Institute, Nedlands, Australia; Medical School, University of Western Australia, Crawley, Australia; Department of Infectious Diseases, Perth Children's Hospital, Nedlands, Australia; Wesfarmers Centre of Vaccines and Infectious Diseases, Telethon Kids Institute, Nedlands, Australia; Medical School, University of Western Australia, Crawley, Australia; Menzies School of Health Research, Charles Darwin University, Darwin, Australia; Department of Infectious Diseases, Perth Children's Hospital, Nedlands, Australia

**Keywords:** households, *Streptococcus pyogenes*, transmission, infectious disease, systematic review

## Abstract

**Background:**

Although *Streptococcus pyogenes* (Strep A) is the sixth-most common infectious disease globally, its transmission within the household remains an understudied driver of infection. We undertook a systematic review to better understand the transmission of Strep A among people within the home, while highlighting opportunities for prevention.

**Methods:**

A search strategy was applied to 5 databases between September 2022 and March 2023. Results were limited to articles published between January 2000 and March 2023. Texts were reviewed by 2 authors and the following data extracted: article details (title, author, year), study type, transmission year, country, participant age, infection status, molecular testing, and transmission mode. Funding was provided by the Australian National Health and Medical Research Council (GNT2010716).

**Results:**

The final analysis comprised 28 texts. Only 7 studies (25.0%) provided sufficient detail to identify the Strep A transmission mode: contact (n = 4), vehicle (bedding, clothing, other fabric, and medical equipment; n = 2), and contact with animals (n = 1). All others were classified as household (specific mode unascertainable). Most articles reported outbreaks involving invasive Strep A infections.

**Conclusions:**

There is limited literature regarding household transmission of Strep A. Understanding transmission in this setting remains imperative to guide control methods.

## Teaser

By taking a systematic approach, we attempted to describe the primary mechanisms of strep A person-to-person transmission within households. Household transmission of Strep A is poorly described in the literature with only 7 studies (25.0%) providing sufficient detail to appropriately infer the transmission mode.

Group A *Streptococcus* (hereafter, Strep A) is a bacterium responsible for significant global morbidity and mortality [1], causing an array of illnesses: superficial (ie, pharyngitis, impetigo), invasive (ie, bacteremia, sepsis), and toxin mediated (ie, scarlet fever) [2]. An estimated 162 million cases of Strep A impetigo are present globally at any one time, with 616 million annual cases of pharyngitis and 517 000 deaths caused by invasive Strep A infections per year worldwide [1, 3]. Strep A also has the potential to cause postinfectious immune-mediated illnesses—acute rheumatic fever (ARF) and acute poststreptococcal glomerulonephritis—which can progress to causing rheumatic heart disease and chronic kidney disease, respectively [1]. Susceptibility to postinfectious sequelae is driven by recurrent Strep A infection of the skin or throat in early life, leading to immune priming and vulnerability to these illnesses [3]. There are >200 known strains of Strep A, with a predilection for different clinical manifestations and evoking different host immune responses.

At a population level, incidence of Strep A infection and sequelae has long been associated with the consequences of socioeconomic marginalization, including poverty and household crowding [4–6]. Contemporary case-control data from Aotearoa New Zealand strengthens the case for household crowding as a driver of Strep A infections [7, 8]. This is consistent with epidemiologic correlations in Australia, with the burden of Strep A and sequelae highest among remote-living Aboriginal and Torres Strait Islander people in settings of poor household construction and limited infrastructure maintenance [9–11]. Despite these associations, there has been little exploration of how household factors contribute to increased Strep A risk and what mitigation strategies may be possible. Understanding infectious disease transmission within houses and households offers important opportunities to understand and address disease effect [12, 13]. This systematic review scopes what is published about Strep A transmission events within household settings to inform thinking about environmental health responses in Australia.

## METHODS

This review was completed according to the PRISMA statement (Preferred Reporting Items for Systematic Reviews and Meta-analyses) [14] (supplementary material). To better understand the transmission of Strep A among people within the home and thus identify priority opportunities for effective prevention at the household level, we undertook a systematic review with 2 aims: first, identify the most frequent mechanisms of transmission in the household; second, describe the mechanisms of Strep A transmission while highlighting opportunities for prevention [15]. This review complements a formal systematic review of Strep A transmission in all contexts inclusive of hospital settings [13].

### Search Strategy

The search was conducted across 5 databases (Medline, Embase, PubMed, Web of Science, and Scopus) between August 2022 and March 2023 and can be viewed as part of the supplementary material. Where necessary, Medical Subject Headings terms were used as an adjunct to keywords. One author (S. L. E.) completed title and abstract screening and selected articles according to the following inclusion criteria: studies investigating an outbreak or transmission of Strep A in a household, studies of households that postulated a possible mode of transmission, and articles available in English. Prior work investigating Strep A transmission in all settings [13] demonstrated household studies undertaken from 2000 onward to be more in-depth and conducive to answering the research question. On this basis, only studies published between January 2000 and March 2023 were included. There were no limitations on study designs, participant age, geographic setting, or participant health. Narrative reviews, laboratory-based research, studies detailing infection trend, research on animal disease, or those addressing diagnosis and treatment were excluded. Given the likelihood of nosocomial transmission in nursing homes or residential care, studies detailing outbreaks in these settings were excluded. Studies related to in-home nursing or health care were also excluded, as there are substantial differences between these and households, such as number of residents, contact with staff, requirement for large numbers of staff, and air handling. References from all included studies were screened to capture any studies not identified in the search strategy.

All selected texts were reviewed by 2 authors (S. L. E., E. P.) and the following data extracted: article details (title, author, year), type of study, year of transmission, country, geography (urban, rural, remote), participant age, infection status (exposed, symptomatic, asymptomatic), molecular testing, and mode of transmission. Strep A infection was classified as follows: superficial (eg, pharyngitis, impetigo, scarlet fever), severe (isolation of Strep A from a nonsterile site in combination with symptoms attributable to streptococcal toxic shock syndrome), or invasive (isolation of Strep A from a normally sterile site in combination with severe clinical presentation). In each study, the “index case” was noted as the first case requiring medical attention. Discrepancies between authors were discussed and amended accordingly. Notes were also taken where authors hypothesized possible drivers of transmission within the household. The primary outcome was the mechanism of Strep A transmission within the home. The secondary outcome was an assessment of the modes of transmission within the same setting with potential opportunities for prevention. Descriptive analyses were used to report the frequency of variables across the cohort of studies, and all other results were analyzed narratively.

## RESULTS

### Search Strategy

**Figure 1.**
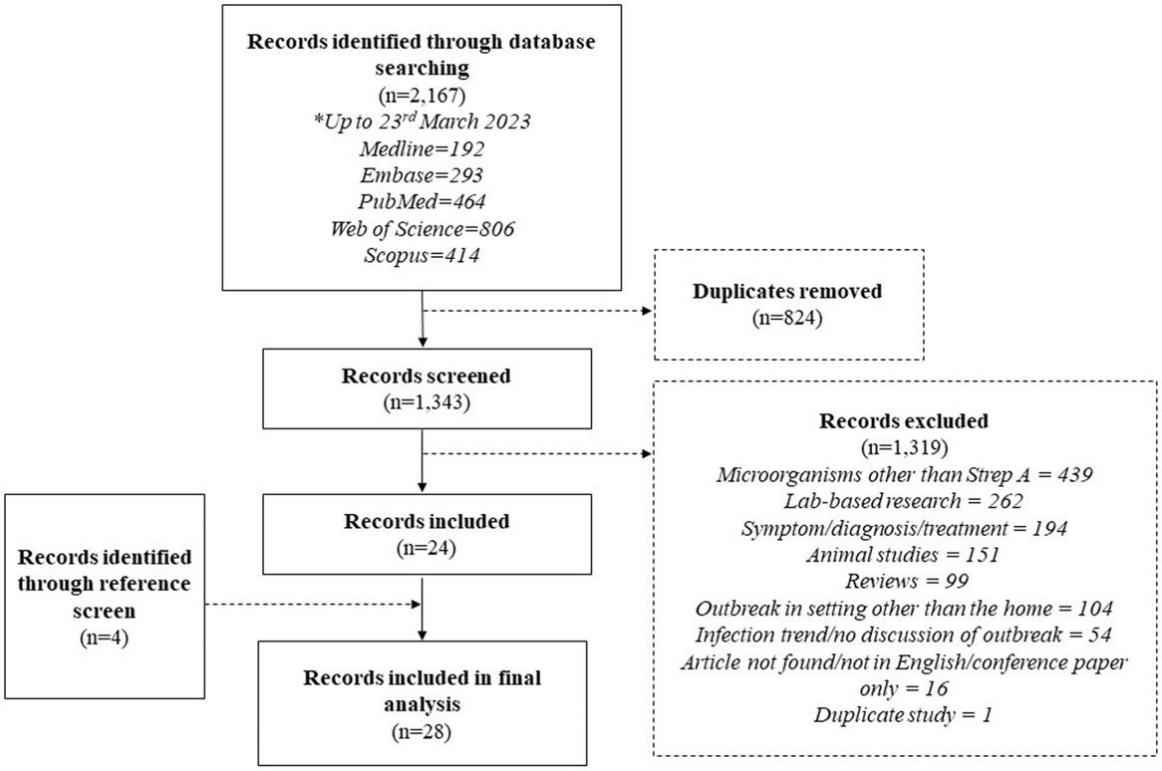
Flow diagram of search results and screening process.

A total of 2167 articles were retrieved, with 1343 remaining after the removal of duplicates. Following title and abstract screening against the inclusion and exclusion criteria, 24 articles were included in the analysis. Reference screening identified 4 articles eligible for inclusion, and 28 articles composed the final review. The search strategy and primary reasons why articles were excluded are presented in Figure 1. The studies and primary findings are summarized in Table 1.

**Table 1.** Summary of Included Articles

Study	Proposed Transmission Route	Age of Index Case, y	Infection Type in Index Case	Age of Infected Contacts, y	Infection Type in Contacts
Barnham (2001) [16]	Household contact not specified	86	Necrotizing fasciitis secondary to skin trauma	32, 87, 88	Fatal pneumonia, pharyngeal abscess
Husain (2001) [17]	Household contact not specified	4	Invasive Strep A infection (bacteremia and arthritis)	7	Invasive Strep A infection (anatomic focus not stated)
Huang (2001) [18]	Household contact not specified	4	Streptococcal toxic shock syndrome	5, 6, 8	Streptococcal toxic shock syndrome, asymptomatic pharyngeal carriage
Gisser (2002) [19]	Contact (sexual)	45	Peritonitis and toxic shock syndrome	1 contact, age unknown	Asymptomatic pharyngeal carriage
Manalo (2002) [20]	Contact (sexual)	26	Tuboovarian abscesses	1 contact, age unknown	Pharyngitis
Smolyakov (2002) [21]	Vehicle (medical equipment, intravenous drugs)	38	Necrotizing fasciitis (left arm, right leg)	32	Necrotizing fasciitis (left calf)
Recco (2002) [22]	Household contact not specified	77	Invasive Strep A infection	74	Invasive Strep A infection
Ip (2003) [23]	Household contact not specified	10	Pneumonia and streptococcal toxic shock syndrome	77	Pneumonia and streptococcal toxic shock syndrome, pharyngitis
Laustrup (2003) [24]	Contact (skin-to-skin)	60	Invasive Strep A infection with necrotizing fasciitis secondary to skin trauma	51	Necrotizing fasciitis secondary to skin trauma
Robinson (2003) ^a^ [25]	Household contact not specified	0, 76; 523 other cases, ages unknown	Invasive Strep A infection (only cellulitis and bacteremia specified)	39, 69; 61 other contacts, ages unknown	Pharyngitis, cutaneous infection, pneumonia, cellulitis, necrotizing fasciitis
Mazón 2003 [26]	Household contact not specified	1 case, age unknown	Pharyngitis	4 contacts, ages unknown	Pharyngitis, perianal dermatitis, asymptomatic pharyngeal carriage (repeated episodes)
Roy 2003 [27]	Household contact not specified	46	Pneumonia and empyema	3, 7, 9, 10, 12, 14	Pneumonia, empyema, sepsis, pleural effusion, pharyngitis
Lindbaek (2004) ^a^ [28]	Household contact not specified	110 cases, ages unknown	Pharyngitis	40 contacts, ages unknown	Pharyngitis
Green (2005) [29]	Contact (sexual)	36	Septicemia	34	Septicemia
Sobel (2007) [30]	Vehicle (bedding, clothing, and other fabric)	39, 42 (2 case reports)	Vulvovaginitis	2 contacts, ages unknown	Perianal carriage
Hansen (2007) [31]	Household contact not specified	4	Vulvovaginitis and pharyngitis	1 contact, age unknown	Pharyngitis
Martinaud (2010) [32]	Household contact not specified	87	Streptococcal toxic shock syndrome	16, 47, 82	Pharyngitis, streptococcal toxic shock syndrome, retroperitoneal abscess
Torres (2016) [33]	Household contact not specified	7	Necrotizing fasciitis (right thigh) and streptococcal toxic shock syndrome	5, 6, 29	Recent pharyngitis, asymptomatic pharyngeal carriage
Middleton (2014) [34]	Household contact not specified	14 wk	Invasive Strep A peritonitis and streptococcal toxic shock syndrome	14 wk (twin)	Pneumonia and streptococcal toxic shock syndrome
Duployez (2017) [35]	Household contact not specified	67	Necrotizing fasciitis (leg)	66; 2 other contacts, ages unknown	Necrotizing fasciitis (right arm), recent pharyngitis
Mearkle (2017) [36]	Household contact not specified	24 cases, ages unknown	Invasive Strep A infection	24 contacts, ages unknown	Invasive Strep A infection
Flores (2017) [37]	Household contact not specified	3.5	Invasive Strep A infection	1.5, 2.5, 5	Invasive Strep A infection, pharyngitis,
Doyon (2017) [38]	Household contact not specified	4	Orbital cellulitis	4, 7	orbital cellulitis, asymptomatic pharyngeal carriage
Veraldi (2018) [39]	Contact with animal	68	Severe skin infection following trauma (cat scratch)	…	No contacts
Watts (2019) [40]	Household contact not specified	11 cases, ages unknown	Scarlet fever	11 contacts, ages unknown	Invasive Strep A infection
Adebanjo (2020) ^a^ [41]	Household contact not specified	24, 36, 42, 49, 53, 70, 77, 78, 91, 92	Cellulitis, chorioamnionitis, septic shock, abscess, pneumonia, bacteremia	0, 48, 53, 54, 67,69, 72, 82, 85, 89 95	Bacteremia, cellulitis, meningitis, septic shock, abscess, septic arthritis
Álvarez (2020) [42]	Household contact not specified	64	Pharyngitis, invasive Strep A infection	1 contact, age unknown	Invasive Strep A infection
Markowitz (2022) [43]	Household contact not specified	40	Necrotizing fasciitis (right arm)	40	Cellulitis, necrotizing fasciitis (right arm)

^a^Study involved population-based surveillance data or large household cohort.

### Study Design

Of the 28 articles in the review, 20 (71.4%) were case reports and 8 (28.6%) were cohort studies. Most outbreaks were from Europe (n = 12, 42.8%), with 11 (39.2%) from North America, 4 (14.3%) from Asia, and 1 each from Oceania and South America. Too few articles reported on seasonality, geography of outbreak occurrence, or participant income to report meaningful descriptive statistics for these parameters. Comments were also unable to be made regarding population marginalization or vulnerability—important considerations given the prevalence of Strep A infections in resource-limited settings.

### Population Demographics

Every article reported at least some demographics of cases and contacts, although the level of detail varied. Most studies included a mix of age groups (n = 12, 43.8%), with 5 (17.9%) describing only those aged <18 years, 7 (25.0%) aged 18 to 60 years, and 4 (14.3%) >60 years. At least 1 special health condition in cases prior to Strep A infection was described in 18 (64.3%) outbreaks; these were classified as multiple complications (>1 identified health challenge; n = 9, 50.0%), recent history of infectious disease (n = 5, 27.8%), acute injury/postoperative (n = 3, 16.7%), and prematurity (n = 1, 5.6%). The rest were previously healthy (n = 6, 21.4%), or insufficient detail was provided to appropriately classify (n = 4, 14.3%).

### Disease

Most articles reported on >1 Strep A disease manifestation among people in each household outbreak (n = 19, 65.5%). Invasive Strep A infection was present in 21 (72.4%) studies, severe disease in 15 (n = 51.7%), and superficial skin and/or throat infections in 15 (51.7%). Asymptomatic carriage was also noted in 6 studies (20.7%). No instances of postinfectious immune-mediated sequelae were cited in any studies.

### Transmission

Only 7 studies (25.0%) provided sufficient detail to appropriately identify the mode of Strep A transmission. These were contact (n = 4), vehicle (bedding, clothing, and other fabric and medical equipment; n = 2), and contact with animals (n = 1). All others were classified as household (specific mode unascertainable).

### Contact Transmission: Including Sexual Transmission and Skin-to-Skin Contact

Of the 4 studies in this section, 3 undertook molecular testing to identify the source of infection [19, 20, 24]. Gisser et al [19] described a case of peritonitis in a previously healthy woman with an intrauterine device (IUD). Identical strains were isolated in the culture of a throat specimen from the patient's male partner (asymptomatic) and the patient's IUD. The authors postulated that minor trauma caused by the IUD placement a year earlier facilitated passage of the bacteria across the endometrium, with the most likely transport route of Strep A being oral sex. Manalo et al [20] reported a case of orogenital transmission causing a Strep A tubo-ovarian abscess. The patient's partner had a mild respiratory tract infection 1 month prior, and the couple indicated engaging in oral sex prior to the infection. Organisms isolated from the patient's peritoneal swab specimen and her partner's throat culture had identical electrophoretic patterns. Oral sex was postulated by Green et al [29] to underpin Strep A transmission in a couple who had streptococcal septicemia; however, the specific source could not be determined. While the woman had an IUD, no organisms grew from swabs taken from it, possibly due to preceding antibiotics, and throat swabs were not collected.

Laustrup et al [24] described a case of invasive Strep A infection causing necrotizing fasciitis transmitted between a cohabiting couple. One partner sustained a soft tissue injury that became infected; the other incurred a small laceration several days later. Direct contact via wound care from 1 partner to another preceded both being diagnosed with invasive Strep A infection. The same strain of Strep A was determined upon isolation via polymerase chain reaction of DNA that was recovered from wound swabs.

### Vehicle Transmission

Sobel et al [30] reported 2 cases of recurrent Strep A vulvovaginitis in women whose male partners were identified as gastrointestinal carriers of Strep A. Molecular typing of samples revealed that each couple had identical Strep A strains, with no other household contacts returning positive cultures. The authors postulated perianal shedding with contamination of bedding as the mechanism for transmission.

Smolyakov et al [21] reported the simultaneous development of streptococcal necrotizing fasciitis in limbs of a cohabitating couple with a history of intravenous heroin injection. Molecular typing (*emm* and T antigens) of blood, synovial fluid, and wound cultures identified identical strains. No throat cultures were completed. History confirmed the regular sharing of injecting equipment, which was postulated as the most likely transmission route.

### Contact With Animal

Strep A is commonly described as a human-only pathogen; thus, animals are not considered sources of transmission [44]. However, Veraldi and Minuti [39] reported an incident of severe streptococcal skin infection in an elderly woman following a cat scratch. While molecular typing was not undertaken, Strep A was isolated from the patient's wound as well as the cat's oral cavity and claws, and it was inferred that the cat was potentially the source of human infection.

### Household: Specific Mode Unascertainable

All remaining studies (n = 21, 75.0%) provided insufficient detail to adequately determine the specific mode of transmission within households, though noting close contact with an index case and subsequently infected family members to be the primary risk factor. However, it could not be ascertained whether droplet, skin-to-skin contact, or another mode was responsible. Of these, strains between the index case and contacts were identical in 15 (71.4%) of investigated outbreaks, indicating definite Strep A transmission among household contacts. The remaining studies did not report on molecular typing results; hence, only assumptions were provided. The majority (n = 17, 80.9%) detailed outbreaks involving people aged <18 years. Twelve studies (41.4%) reporting cases of invasive or severe Strep A disease noted at least 1 contact who had a previous superficial Strep A infection that had cleared symptomatically and/or a close contact who was identified as an asymptomatic carrier.

A handful of studies warrant noting. Lindbaek et al [28] observed a significant relationship between spread of superficial Strep A infection (pharyngitis) within the household and the presence of children—postulated to be a result of closer contact among household members where children are present, especially when children are ill. Watts et al [40] found infants and contacts aged ≥75 years to be at highest risk for invasive Strep A infections within 60 days of their household experiencing a scarlet fever case (60-day incidence rate, 35.3 cases/100 000 person-years; 12.2 times higher than the background rate of 2.89 cases). No specific transmission modes were confirmed.

Mearkle et al [36] assessed the risk of invasive Strep A infections from household contacts (United Kingdom) and identified the 30-day incidence rate to be 4520 cases/100 000 person-years, 1940 times higher than the background incidence (2.34 cases/100 000 person-years). While no specific transmission modalities were postulated, risk factors included being a mother in the postnatal period, being a neonate, having a higher household occupancy size, and being in a spousal relationship and aged ≥75 years. In a US study, Adebanjo et al [41] reported the 30-day incidence rate of household Strep A transmission as 1240 cases/100 000 person-years among all ages and 4122 cases/100 000 person-years in an older subgroup in which the age of the secondary case was ≥65 years.

No other studies reported information adequate to infer risk factors or identify traits of nonpatients that may have been protective against illness.

## DISCUSSION

We attempted to describe the primary mechanisms of Strep A transmission within households using a systematic approach. As evidenced by the results, there is a notable lack of studies reporting on this topic and providing adequate detail to form definitive conclusions despite the heavy burden of infection experienced globally from this pathogen. Hence, inferences regarding transmission modalities cannot be discerned.

An assessment of the literature regarding Strep A outbreak investigations in other environments demonstrated an application of more thorough epidemiologic methods, allowing for accurate discernment of transmission mechanisms. This tends to be more common in settings where the risk of nosocomial transmission is heightened, especially among medically vulnerable populations in clinical environments. For example, Mahida et al [45] identified perianal shedding from a health care worker (HCW) to be the source of an outbreak in an elderly care medical ward through HCW screening and environmental sampling using swabs and settle plates. In this outbreak, an upholstered chair on the ward used by infected patients and the asymptomatic HCW was the most likely source of transmission. Strep A dissemination by small airborne particles on postoperative wards were discussed by Mastro et al [46] and Berkelman et al [47], where in each example an asymptomatic operating nurse was considered to be the source, one colonized on the scalp and the other vaginally with strains that matched the infection strain. Cordery et al [48] demonstrated the usefulness of swabbing and environmental sampling to assess the transmission of Strep A in school classrooms amid upsurges in scarlet fever rates, indicating the applicability of expanded methods beyond clinical settings.

In the articles in our review, limited environmental sampling took place within the household. The household provides a valuable setting to investigate Strep A transmission by asymptomatic and symptomatic members, and the results from this review indicate the need for prospective screening studies in high-risk household environments inclusive of regular personal and environmental sampling. Based on the findings here and from investigation in health care settings, personal sampling should include throat, skin, rectal, and vaginal swabs, and environmental sampling should include bedsheets and soft furnishings for investigation of an invasive Strep A case. Sampling for superficial Strep A transmission may be simplified to throat and skin swabs, with soft and hard environmental surfaces also swabbed. Considerations to acceptability of such invasive swabbing within households must be made.

In our study, which was targeted at household transmission, we identified only 1 article reporting transmission events likely originating from superficial infections [28]. Severe and invasive Strep A infections were most often reported, given the clinical severity of such infections and the known likelihood of secondary cases among households [36]. This prominence may be due to these diseases being notifiable and the assumption that contacts infected with the same strain will have a similar (invasive) clinical presentation, which invokes a greater clinical response occasionally involving epidemiologic investigation. However, superficial infections such as impetigo and pharyngitis are far from benign, with the potential for progression to poststreptococcal sequelae if untreated. Investigation to understand the household transmissibility of Strep A pharyngitis and impetigo is needed in regions experiencing high rates of ARF, rheumatic heart disease, and acute poststreptococcal glomerulonephritis to inform preventative public health measures and interventions. From a post hoc molecular analysis of skin and throat isolates collected in a household study of individuals at risk of ARF, asymptomatic throat carriers were recently hypothesized as reservoirs for Strep A strains that cause impetigo in high-burden remote northern Australian Aboriginal community settings [9]. This was based on the finding that 63% of household transmission events of superficial Strep A infections were connected to the detection of the same isolates of Strep A in the throat of asymptomatic individuals prior to it being detected from a skin infection. These highlight occult respiratory spread as a potential transmission factor in households in this setting.

Few articles specified a mode of Strep A transmission. Of those that did, orogenital sex was indicated as a likely source in 3 instances. While each case study was well reported and investigated, it is unlikely that this mode is a significant contributor to global disease burden when compared with other means: droplet, contact, or environmental. We hypothesize that the nascent and novel nature of each case drove the desire to investigate and publish. The case study involving a cat scratch should also be treated as novel and rare, given that there are few similar reports in the literature of animal-to-human transmission of Strep A.

Almost all articles discussed the importance of providing prophylactic antibiotics to close contacts of invasive Strep A cases; however, this may be impractical as a significant proportion of secondary cases occurred within 1 day of the index [35, 36]. Mearkle et al found that 38% of pairs were co–primary cases or had only 1 day between initial and subsequent infections [36]. Guidelines for the prevention of invasive Strep A infections among known contacts would be strengthened by informed understanding of transmission mechanisms within the household, which remain uncertain.

The literature continues to assess risk factors associated with Strep A infection—mostly the postinfectious sequelae—with great inroads to informing prevention strategies. Oliver et al interviewed 55 individuals with ARF and determined a high likelihood of their experiencing household crowding (58%), bed sharing (49%), dampness and mold (76%), cold (82%), and cohabitation with smokers (71%) [49]. This descriptive study provided the justification for further work, including that completed by Bennett et al [7] and Baker et al [8], which identified crowding to be a risk factor for Strep A pharyngeal and skin infections [7] and ARF [7]. Cannon et al [50] also found that household risk factors for ARF in early and middle childhood included large family size, with overcrowding a risk factor for development of ARF in late childhood. This knowledge of epidemiologic risk factors indicates who requires implementation of prevention strategies to interrupt household transmission of Strep A, but precisely what those prevention strategies should be requires further precise knowledge of transmission pathways of Strep A in households.

This research has several limitations. First, most studies were from Europe and North America, with fewer from other continents. We note this geographic bias affecting the generalizability of results, specifically in resource-limited settings. Generalizability is further affected by the majority of articles being case reports, which are acknowledged as being less robust than other study designs. The overall dearth of data prevented the application of more rigorous statistical methods, allowing only for a narrative analysis.

## CONCLUSION

Household transmission of Strep A is poorly described in the literature. Given the time spent in household environments, a greater understanding of Strep A transmission modalities in this setting is required to guide prevention methods.

## Supplementary Data

Supplementary materials are available at *The Journal of Infectious Diseases* online (http://jid.oxfordjournals.org/). Supplementary materials consist of data provided by the author that are published to benefit the reader. The posted materials are not copyedited. The contents of all supplementary data are the sole responsibility of the authors. Questions or messages regarding errors should be addressed to the author.

## Supplementary Material

jiae136_Supplementary_Data

## Notes

***Author contributions***. S. L. E.: conceptualization, methodology, software, validation, formal analysis, investigation, resources, data curation, writing–original draft, writing–review and editing, visualization, project administration. S. B.: conceptualization, methodology, writing–review and editing. J. D.: methodology, formal analysis, investigation, writing–review and editing. E. P.: formal analysis. H. M. M. T.: writing–review and editing, supervision. R. W.: conceptualization, writing–review and editing, visualization, supervision, funding acquisition. A. M. R.: conceptualization, writing–review and editing, visualization, supervision, funding acquisition. N. L.: conceptualization, methodology, writing–review and editing, visualization, supervision, project administration, funding acquisition. A. P. R.: conceptualization, writing–review and editing, visualization, funding acquisition. J. R. C.: writing–review and editing, visualization, supervision, funding acquisition. A. C. B.: conceptualization, writing–review and editing, visualization, supervision, funding acquisition.

***Disclaimer.*** The funders had no role in study design, data collection and analysis, decision to publish, or preparation of the manuscript.

***Financial support*.** This work was supported by a Research Program Training scholarship at the University of Western Australia, a Wesfarmers Centre of Vaccines and Infectious Diseases Top Up scholarship, and the Stan and Jean Perron Foundation to S. L. E.; a Higher Degree by Research Scholarship at the University of Queensland to S. B.; National Health and Medical Research Investigator Awards (GNT1173874 and GNT1175509 to J. R. C. and A. C. B., respectively); and an National Health and Medical Research Synergy grant (GNT2010716).
