# Supplementary material for: Systematic Review of Household Transmission of Strep A: A Potential Site for Prevention That Has Eluded Attention
Source: J Infect Dis. 2024 Mar 13;230(4):e798–806. doi: 10.1093/infdis/jiae136 (PMC11481456; doi:10.1093/infdis/jiae136)
Supplement: jiae136_Supplementary_Data [file jiae136_supplementary_data.pdf]

| Section and Topic | Item # | Elements recommended for reporting |
|---|---|---|
| **TITLE** | | |
| TITLE | 1 | • Systematic review of household transmission of Strep A: a potential site for prevention that has eluded dedicated attention. |
| **ABSTRACT** | | |
| ABSTRACT | 2 | • Background: Transmission of Streptococcus pyogenes (Strep A) within the household remains an understudied driver of infection. We undertook a systematic review to better understand the transmission of Strep A between people within the home to describe the direct mechanisms of Strep A transmission while highlighting opportunities for prevention.<br>• Methods: A search strategy was applied to five databases between September 2022 and March 2023 according to the following inclusion criteria: studies investigating an outbreak of or transmission of Strep A in a household; studies of households that postulated a possible mode of transmission; and manuscripts available in English. Results were limited to those published between January 2000 and March 2023. Texts were reviewed by one author and the following data extracted: article details (title, author, year), type of study, year of transmission, country, geography (urban, rural, remote), participant age, infection status (exposed, symptomatic, asymptomatic), molecular testing and mode of transmission. Funding was provided by the Australian National Health and Medical Research Council (NHMRC, grant number GNT2010716).<br>• Results: The final analysis comprised 28 texts. Only seven studies (25.0%) provided sufficient detail to appropriately identify the mode of Strep A transmission. These were contact (4), vehicle (bedding, clothing, and other fabric, and medical equipment, 2), and vector (animals, 1). All others were classified as household (specific mode unascertainable). Most articles reported outbreaks involving invasive Strep A infections.<br>• Conclusions: There is limited literature regarding household transmission of Strep A, and understanding transmission in this setting to guide control methods remains imperative. |
| **INTRODUCTION** | | |
| RATIONALE | 3 | • At a population level, incidence of Strep A infection and sequalae have long been associated with the consequences of socioeconomic marginalisation, including poverty and household crowding. Contemporary case-control data from Aotearoa New Zealand strengthens the case for household crowding as a driver of Strep A infections. This is consistent with epidemiologic correlations in Australia, with the burden of Strep A and sequalae highest among remote living Aboriginal and Torres Strait Islander people in settings of poor household construction and limited infrastructure maintenance. Despite these associations, there has been little exploration of how household factors contribute to increased Strep A risk and what mitigation strategies may be possible. Understanding infectious disease transmission within houses and households offers important opportunities to understand and address disease effect. This review scopes what is published about Strep A transmission events within household settings to inform thinking about environmental health responses in Australia. |
| OBJECTIVES | 4 | • Identify the most frequent mechanisms of transmission in the household.<br>• Describe the mechanisms of Strep A transmission while highlighting opportunities for prevention. |
| **METHODS** | | |
| ELIGIBILITY CRITERIA | 5 | • Studies involving children, adolescents, and adults regardless of age, gender, health conditions, or other sociodemographic characteristics.<br>• All study designs (including but not limited to case series, outbreak investigations, cross-sectional, cohort and case-control studies) investigating modes of transmission of Strep A to humans resulting in human Strep A infection (both symptomatic disease with clinical relevance to Strep A infection and asymptomatic carriage).<br>• Studies that proposed a mode of transmission, regardless of use of molecular typing to confirm the mode.<br>• Studies that investigate transmission or disease acquisition within a household setting.<br>• Studies published in English between January 2000 and August 2022. |
| INFORMATION | 6 | • Keywords used in this search are included elsewhere.<br>• A collection of MeSH Terms and title/abstract/keyword searches were undertaken. The searches were restricted to studies published in English between 2000 and 2022. We obtained our data from the following resources:<br>    • For published studies: PubMed, Scopus, EMBASE, Web of Science, and Medline and Google Scholar.<br>    • For citations: Scopus, EMBASE, Web of Science, and CINAHL.<br>    • For grey literature: WHO IRIS library database, Trove, Research Data Australia, the Grey Literature Report, and Australian Infection Prevention and Control.<br>    • For clinical trial registries: Cochrane Central Register of Clinical Trials, WHO International Clinical Trials Registry Platform, Australian New Zealand Clinical Trial Registry, and ClinicalTrial.gov.<br>• We also searched the reference lists of peer-reviewed articles for published and unpublished materials cited in study reports included in the systematic review, or references cited in systematic review reports on the same or similar topic. |

| Section and Topic | Item # | Elements recommended for reporting | *Strep A transmission in households - PRISMA* |
|---|---|---|---|
| SEARCH STRATEGY | 7 | • Ovid MEDLINE databases were searched on 26 August 2022. | |

| | | |
|---|---|---|
| 1 | Streptococcal infections/ or Streptococcus pyogenes/ | 41276 |
| 2 | (beta haemolytic streptococcus group a or beta hemolytic streptococcus group a or group a beta hemolytic streptococcus or group a beta haemolytic streptococcus or group a strep infection or group a streptococcal infection or group a streptococcal infections or group a streptococcal infections or group A streptococcus or infection, streptococcal or infections, streptococcal or Strep a or Streptococcal infection or Streptococcal infection or streptococcal infections or streptococcal infections or streptococcus a or streptococcus group a, beta haemolytic or streptococcus group a, beta hemolytic or Streptococcus pyogenes).mp. | 46895 |
| 3 | Disease Transmission, Infectious/ or Bacterial infections/ or Communicable disease/ | 116188 |
| 4 | (Transmit or Transmission or transmitted or Communicable disease or Contagious or Contagion or Infectious or communicable diseases or disease, communicable or disease, infectious or diseases, communicable or diseases, infectious or infectious disease or infectious diseases).mp. | 1042432 |
| 5 | Home environment/ or Residence characteristics/ or Family/ or Family characteristics/ | 145621 |
| 6 | (Accommodation or Arrangement, living or Arrangements, living or Characteristic, residence or Characteristics, residence or Dwelling or Familial or Family epidemiology or Family member or Family members or Family or Household contact or Household contacts or Household or Inter-familial or Intra-familial or Living arrangement or Living arrangements or Lodging or Quarters or Residence characteristic or Residence characteristics or Residence or Settlement or Shelter).mp. | 1422869 |
| 7 | 1 or 2 | 46895 |
| 8 | 3 or 4 | 1104051 |
| 9 | 5 or 6 | 1422999 |
| 10 | 7 and 8 and 9 | 288 |
| 11 | limit 10 to yr="2000 -Current" | 188 |

- MEDLINE databases were searched on 23 March 2023.

| | | |
|---|---|---|
| Streptococcal infections/<br>Streptococcus pyogenes/<br>beta haemolytic streptococcus group a.mp<br>beta hemolytic streptococcus group a.mp<br>group a beta hemolytic streptoquarterccus.mp<br>group a beta haemolytic streptococcus.mp<br>group a strep infection.mp<br>group a streptococcal infection.mp<br>group a streptococcal infections.mp<br>group a streptococcal infections.mp<br>group A streptococcus.mp<br>infection, streptococcal.mp<br>infections, streptococcal.mp<br>Strep a.mp<br>Streptococcal infection.mp<br>Streptococcal infection.mp<br>streptococcal infections.mp<br>streptococcal infections.mp<br>streptococcus a.mp<br>streptococcus group a, beta haemolytic.mp<br>streptococcus group a, beta hemolytic.mp<br>Streptococcus pyogenes.mp<br>• | Disease Transmission, Infectious/<br>Bacterial infections/<br>Communicable disease/<br>Transmit.mp<br>Transmission.mp<br>transmitted.mp<br>Communicable disease.mp<br>Contagious.mp<br>Contagion.mp<br>Infectious.mp<br>communicable diseases.mp<br>disease, communicable.mp<br>disease, infectious.mp<br>diseases, communicable.mp<br>diseases, infectious.mp<br>infectious disease.mp<br>infectious diseases.mp | Home environment/<br>Residence characteristics/<br>Family/<br>Family characteristics/<br>Accommodation.mp<br>Arrangement, living.mp<br>Arrangements, living.mp<br>Characteristic, residence.mp<br>Characteristics, residence.mp<br>Dwelling.mp<br>Familial.mp<br>Family epidemiology.mp<br>Family member.mp<br>Family members.mp<br>Family.mp<br>Household contact.mp<br>Household contacts.mp<br>Household.mp<br>Inter-familial.mp<br>Intra-familial.mp<br>Living arrangement.mp<br>Living arrangements.mp<br>Lodging.mp<br>Quarters.mp<br>Residence characteristic.mp<br>Residence characteristics.mp<br>Residence.mp<br>Settlement.mp<br>Shelter.mp |

- Embase databases were searched on 23 March 2023.

| | | |
|---|---|---|
| group a streptococcal infection/<br>Streptococcus group A/<br>streptococcus pyogenes/<br>beta haemolytic streptococcus group a.mp<br>beta hemolytic streptococcus group a.mp<br>group a beta hemolytic streptococcus.mp<br>group a beta haemolytic streptococcus.mp<br>group a strep infection.mp<br>group a streptococcal infection.mp<br>group a streptococcal infections.mp<br>group a streptococcal infections.mp<br>group A streptococcus.mp<br>infection, streptococcal.mp<br>infections, streptococcal.mp<br>Strep a.mp<br>Streptococcal infection.mp<br>Streptococcal infection.mp<br>streptococcal infections.mp<br>streptococcal infections.mp<br>streptococcus a.mp<br>streptococcus group a, beta haemolytic.mp<br>streptococcus group a, beta hemolytic.mp<br>Streptococcus pyogenes.mp | disease transmission/<br>disease transmission, infectious/<br>disease transmission via medicinal product/<br>communicable disease/<br>communicable disease control/<br>communicable disease.mp<br>communicable diseases.mp<br>contagion.mp<br>contagious.mp<br>disease transmission.mp<br>disease, communicable.mp<br>disease, infectious.mp<br>diseases, communicable.mp<br>diseases, infectious.mp<br>infection transfer.mp<br>infection transmission.mp<br>infectious disease transmission.mp<br>infectious disease.mp<br>infectious diseases.mp<br>Infectious.mp<br>transmission of infection.mp<br>transmission of infectious disease.mp<br>Transmission.mp<br>Transmit.mp<br>transmitted.mp | Home/<br>Family/<br>Home environment/<br>residence characteristic/<br>built environment/<br>housing/<br>Accommodation.mp<br>Arrangement, living.mp<br>Arrangements, living.mp<br>Characteristic, residence.mp<br>Characteristics, residence.mp<br>Dwelling.mp<br>Familial.mp<br>Family epidemiology.mp<br>Family member.mp<br>Family members.mp<br>Family.mp<br>Home.mp<br>House.mp<br>Household contact.mp<br>Household contacts.mp<br>Household.mp<br>housing.mp<br>Inter-familial.mp<br>Intra-familial.mp<br>living arrangement.mp<br>Living arrangements.mp<br>Lodging.mp<br>Residence characteristic.mp<br>Residence characteristics.mp<br>Residence.mp<br>Settlement.mp<br>Shelter.mp<br>Quarters.mp |

- Ovid MEDLINE databases were searched on 26 August 2022.

| | | |
|---|---|---|
| 1 | group a streptococcal infection/ or Streptococcus group A/ or streptococcus pyogenes/ | 25176 |
| 2 | (beta haemolytic streptococcus group a or beta hemolytic streptococcus group a or group a beta hemolytic streptococcus or group a beta haemolytic streptococcus or group a strep infection or group a streptococcal infection or group a streptococcal infections or group a streptococcal infections or group A streptococcus or infection, streptococcal or infections, streptococcal or Strep a or Streptococcal infection or Streptococcal infection or streptococcal infections or streptococcal infections or streptococcus a or streptococcus group a, beta haemolytic or streptococcus group a, beta hemolytic or streptococcus pyogenes).mp. | 30324 |
| 3 | disease transmission/ or disease transmission, infectious/ or disease transmission via medicinal product/ or communicable disease/ or communicable disease control/ | 143172 |
| 4 | (communicable disease or communicable diseases or contagion or contagious or disease transmission or disease, communicable or disease, infectious or diseases, communicable or diseases, infectious or infection transfer or infection transmission or infectious disease or transmission or infectious disease or infectious diseases or Infectious or transmission of infection or transmission of infectious disease or Transmission or Transmit or transmitted).mp. [mp=title, abstract, heading word, drug trade name, original title, device manufacturer, drug manufacturer, device trade name, keyword heading word, floating subheading word, candidate term word] | 1230581 |
| 5 | Home/ or Family/ or Home environment/ or residence characteristic/ or built environment/ or housing/ | 143548 |
| 6 | (Accommodation or Arrangement, living or Arrangements, living or Characteristic, residence or Characteristics, residence or Dwelling or Familial or Family epidemiology or Family member or Family members or Family or Home or House or Household contact or Household contacts or Household or housing or Inter-familial or Intra-familial or living arrangement or Living arrangements or Lodging or Residence characteristic or Residence characteristics or Residence or Settlement or Shelter or Quarters).mp. | 2223819 |
| 7 | 1 or 2 | 33837 |
| 8 | 3 or 4 | 1230581 |
| 9 | 5 or 6 | 2224746 |
| 10 | 7 and 8 and 9 | 313 |
| 11 | limit 10 to yr="2000 -Current" | 279 |

- Pubmed databases were searched on 26 August 2022.

| Search number | Query | Sort By | Results |
|---|---|---|---|
| 9 | (#1 or #2) AND (#3 or #4) AND (#5 or #6) | from 2000 - 2022 | 452 |
| 8 | (#1 or #2) AND (#3 or #4) AND (#5 or #6) | | 608 |
| 7 | (#1 or #2) AND (#3 or #4) or (#5 or #6) | | 2,284,989 |
| 6 | Accommodation[Text Word] or Arrangement, living[Text Word] or Arrangements, living[Text Word] or Characteristic, residence[Text Word] or Characteristics, residence[Text Word] or Dwelling[Text Word] or Familial[Text Word] or Family epidemiology[Text Word] or Family member[Text Word] or Family members[Text Word] or Family[Text Word] or Home[Text Word] or House[Text Word] or Household contact[Text Word] or Household contacts[Text Word] or Household[Text Word] or housing[Text Word] or Inter-familial[Text Word] or Intra-familial[Text Word] or living arrangement[Text Word] or Living arrangements[Text Word] or Lodging[Text Word] or Quarters[Text Word] or Residence characteristic[Text Word] or Residence characteristics[Text Word] or Residence[Text Word] or Settlement[Text Word] | | 1,760,411 |
| 5 | Family/ or Home environment/ or Residence characteristic/ or built environment/ or housing/ | | 1,932,971 |
| 4 | communicable disease[Text Word] or communicable diseases[Text Word] or contagion[Text Word] or contagious[Text Word] or disease transmission[Text Word] or disease, communicable[Text Word] or disease, infectious[Text Word] or diseases, communicable[Text Word] or diseases, infectious[Text Word] or infection transfer[Text Word] or infection transmission[Text Word] or infectious disease or transmission[Text Word] or infectious disease[Text Word] or infectious diseases[Text Word] or Infectious[Text Word] or transmission of infection[Text Word] or transmission of infectious disease[Text Word] or Transmission[Text Word] or Transmit[Text Word] or transmitted[Text Word] | | 1,522,239 |
| 3 | communicable diseases/ or communicable disease control/ | | 928,373 |
| 2 | streptococcus pyogenes/ | | 18,617 |
| 1 • | beta haemolytic streptococcus group a[Text Word] or beta hemolytic streptococcus group a[Text Word] or group a beta hemolytic streptococcus[Text Word] or group a beta haemolytic streptococcus[Text Word] or group a strep infection[Text Word] or group a streptococcal infection[Text Word] or group a streptococcal infections[Text Word] or group a streptococcal infections[Text Word] or group A streptococcus[Text Word] or infection, streptococcal[Text Word] or infections, streptococcal[Text Word] or Strep a[Text Word] or Streptococcal infection[Text Word] or Streptococcal infection[Text Word] or streptococcal infections[Text Word] or streptococcal infections[Text Word] or streptococcus a[Text Word] or streptococcus group a, beta haemolytic[Text Word] or streptococcus group a, beta hemolytic[Text Word] or Streptococcus pyogenes[Text Word] | | 47,108 |

- Web of Science was searched on 26 August 2022.

| | | |
|---|---|---|
| streptococcus pyogenes or beta haemolytic streptococcus group or beta hemolytic streptococcus group a or group a beta hemolytic streptococcus or group a beta haemolytic streptococcus or group a strep infection or group a streptococcal infection or group a streptococcal infections or group A streptococcus or infection, streptococcal or infections, streptococcal or Strep a or Streptococcal infection or Streptococcal infection or streptococcal infections or streptococcal infections or streptococcus a or streptococcus group a, beta haemolytic or streptococcus group a, beta hemolytic or Streptococcus pyogenes | communicable disease or communicable disease control or communicable diseases or contagion or contagious or disease transmission or disease, communicable or disease, infectious or diseases, communicable or diseases, infectious or infection transfer or infection transmission or Infectious or infectious disease transmission or infectious diseases or infectious disease or Transmission or transmission of infection or transmission of infectious disease or Transmit or transmitted | Accommodation or Arrangement, living or Arrangements, living or built environment or Characteristic, residence or Characteristics, residence or Dwelling or Familial or Family or Family epidemiology or Family member or Family members or Home environment or Home or House or Household contact or Household contacts or Household or housing or Inter-familial or Intra-familial or living arrangement or Living arrangements or Lodging or Quarters or Residence characteristic or Residence characteristics or Residence or Settlement or Shelter |

- Scopus was searched on 26 August 2022.

( TITLE-ABS-KEY ( "streptococcus pyogenes" OR "beta haemolytic streptococcus group A" OR "beta hemolytic streptococcus group a" OR "group a beta hemolytic streptococcus" OR "group a beta haemolytic streptococcus" OR "group a strep infection" OR "group a streptococcal infection" OR "group a streptococcal infections" OR "group A streptococcus" OR "infection, streptococcal" OR "infections, streptococcal" OR "Strep a" OR "Streptococcal infection" OR "streptococcal infections" OR "streptococcus a" OR "streptococcus group a, beta haemolytic" OR "streptococcus group a, beta hemolytic" OR "Streptococcus pyogenes" ) ) AND ( TITLE-ABS-KEY ( "communicable disease" OR "communicable disease control" OR "communicable diseases" OR "contagion" OR "contagious" OR "disease transmission" OR "disease, communicable" OR "disease, infectious" OR "diseases, communicable" OR "diseases, infectious" OR "infection transfer" OR "infection transmission" OR "Infectious" OR "infectious disease transmission" OR "infectious diseases" OR "infectious disease" OR "Transmission" OR "transmission of infection" OR "transmission of infectious disease" OR "Transmit" OR "transmitted" ) ) AND ( TITLE-ABS-KEY ( "Accommodation" OR "Arrangement, living" OR "Arrangements, living" OR "built environment" OR "Characteristic, residence" OR "Characteristics, residence" OR "Dwelling" OR "Familial" OR "Family" OR "Family epidemiology" OR "Family member" OR "Family members" OR "Home environment" OR "Home" OR "House" OR "Household contact" OR "Household contacts" OR "Household" OR "housing" OR "Inter-familial" OR "Intra-familial" OR "living arrangement" OR "Living arrangements" OR "Lodging" OR "Quarters" OR "Residence characteristic" OR "Residence characteristics" OR "Residence" OR "Settlement" OR "Shelter" ) ) AND ( LIMIT-TO ( PUBYEAR , 2022 ) OR LIMIT-TO ( PUBYEAR , 2023 )) OR LIMIT-TO ( PUBYEAR , 2020 ) OR LIMIT-TO ( PUBYEAR , 2019 ) OR LIMIT-TO ( PUBYEAR , 2018 ) OR LIMIT-TO ( PUBYEAR , 2017 ) OR LIMIT-TO ( PUBYEAR , 2016 ) OR LIMIT-TO ( PUBYEAR , 2015 ) OR LIMIT-TO ( PUBYEAR , 2014 ) OR LIMIT-TO ( PUBYEAR , 2013 ) OR LIMIT-TO ( PUBYEAR , 2012 ) OR LIMIT-TO ( PUBYEAR , 2011 ) OR LIMIT-TO ( PUBYEAR , 2010 ) OR LIMIT-TO ( PUBYEAR , 2009 ) OR LIMIT-TO ( PUBYEAR , 2008 ) OR LIMIT-TO ( PUBYEAR , 2007 ) OR LIMIT-TO ( PUBYEAR , 2006 ) OR LIMIT-TO ( PUBYEAR , 2005 ) OR LIMIT-TO ( PUBYEAR , 2004 ) OR LIMIT-TO ( PUBYEAR , 2003 ) OR LIMIT-TO ( PUBYEAR , 2002 ) OR LIMIT-TO ( PUBYEAR , 2001 ) OR LIMIT-TO ( PUBYEAR , 2000 ) ) View less

| Section and Topic | Item # | Elements recommended for reporting |
|---|---|---|
| SELECTION PROCESS | 8 | • One reviewer screened each record (title and abstract). Report how many reviewers screened each record (title/abstract) and each report retrieved, whether multiple reviewers worked independently at each stage of screening or not, and any processes used to resolve disagreements between screeners.<br>• No automation tools were used during the selection process.<br>• No articles necessitated translation. |
| DATA COLLECTION PROCESS | 9 | • Two reviewers independently collected data from each report. Disagreements were amended during discussion.<br>• No automation tools were utilized. |
| DATA ITEMS (outcomes) | 10a | • Information of infection and control: numbers of patients with GAS infected/ detected; types of GAS infections; routes of GAS transmission; disease control measures.<br>• No changes were made to inclusion or definition of the outcome domains. |

| Section and Topic | Item # | Elements recommended for reporting *Strep A transmission in households - PRISMA* |
|---|---|---|
| DATA ITEMS (other | 10b | • Information of studies: study year, study setting (household, school, healthcare setting, or community); study type (observational or interventional); geographical areas (for distinguishing between high- and low-income areas—remote, rural, urban area; developing, developed country);<br>• Information of participants/patients: age; gender; demographics, presence of underlying disease that could influence infection. |
| STUDY RISK OF BIAS ASSESSMENT | 11 | • Risk of bias was assessed by one reviewer using the Critical Appraisal tool from the Joanna Briggs Institute. Each domain was assessed as 'yes' or 'no' with a tally of the number of 'yes' results reflected in the total score. This was not reported in the final manuscript. |
| EFFECT MEASURES | 12 | • No effect measures were measured. |
| SYNTHESIS METHODS (eligibility for synthesis) | 13a | • Synthesis was unable to be discerned due to the narrative nature of the data. |
| SYNTHESIS METHODS (preparing for synthesis) | 13b | • Please refer to the above. |
| SYNTHESIS METHODS (tabulation and graphical methods) | 13c | • The following data was tabulated in the manuscript to demonstrate the following features of each study; proposed route of Strep A transmission within the household, age of index case/s, infection type in index case/s, age of infected contacts and type of infection in each contact. |
| SYNTHESIS METHODS (statistical synthesis methods) | 13d | • Not applicable. |
| SYNTHESIS METHODS (methods to explore heterogeneity) | 13e | • Not applicable. |
| SYNTHESIS METHODS (sensitivity analysis) | 13f | • Not applicable. |
| REPORTING BIAS ASSESSMENT | 14 | • Missing data was reported as unknown and no tools were used to modulate their possible effect on the dataset. |
| CERTAINTY ASSESSMENT | 15 | • Not applicable. |

| Section and Topic | Item # | Elements recommended for reporting |
|---|---|---|
| **RESULTS** | | |
| STUDY SELECTION (flow of studies) | 16a | 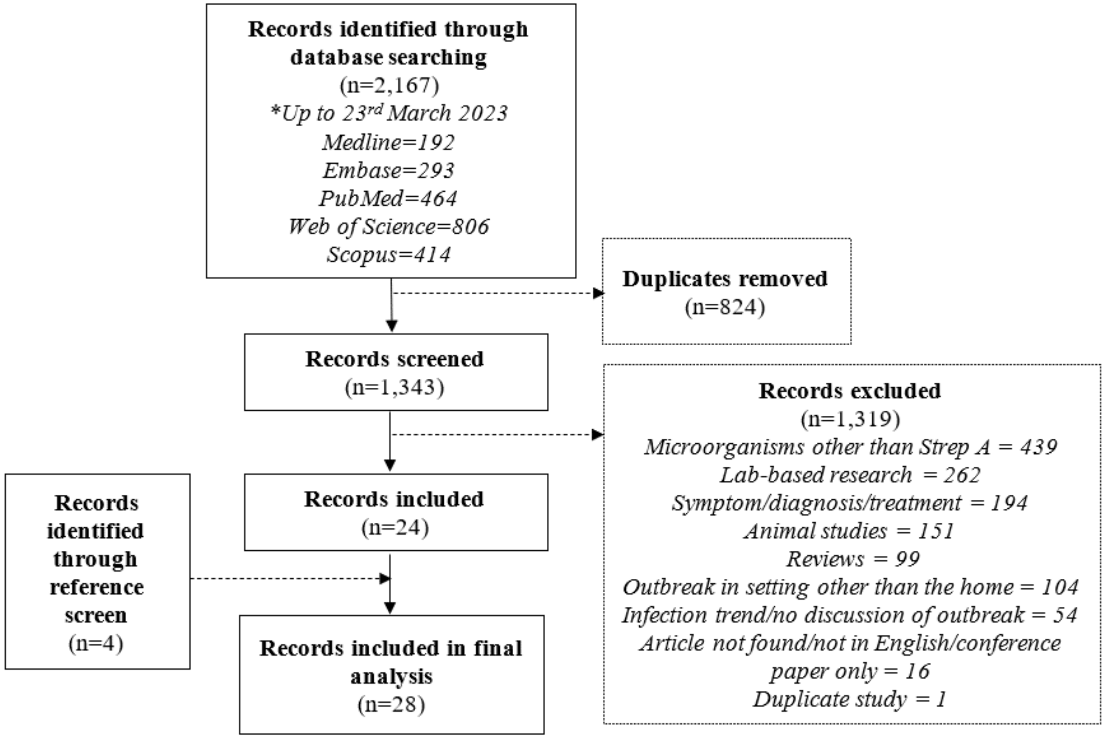 |
| STUDY SELECTION (excluded studies) | 16b | • Please refer to 16b for numbers of and reasons for excluded studies. |
| STUDY CHARACTERISTICS | 17 | • Necessary study characteristics can be viewed in Table 1 of the manuscript. |
| RISK OF BIAS IN STUDIES | 18 | • A risk of bias for each study, while intended to be completed was not due to the narrative style completion of this review and the inadequate quality of each assessed study. |
| RESULTS OF INDIVIDUAL STUDIES | 19 | • Not applicable. |
| RESULTS OF SYNTHESIS (characteristics of contributing studies) | 20a | • Not applicable. |
| RESULTS OF SYNTHESIS (characteristics of statical studies) | 20b | • Not applicable. |
| RESULTS OF SYNTHESIS (results of investigations of heterogeneity) | 20c | • Not applicable statistically, discussed narratively. |

| Section and Topic | Item # | Elements recommended for reporting |
|---|---|---|
| RESULTS OF SYNTHESIS (results of sensitivity analyses) | 20d | • Not applicable. |
| REPORTING BIASES | 21 | • Not applicable. |
| CERTAINTY OF EVIDENCE | 22 | • Not applicable. |
| **DISCUSSION** | | |
| DISCUSSION (interpretation) | 23a | • We attempted to describe the primary mechanisms of Strep A transmission within households using a systematic approach. As evidenced by the results, there is a notable lack of studies both reporting on this topic and providing adequate detail to form definitive conclusions despite the heavy burden of infection experienced globally from this pathogen. Hence inferences regarding transmission modalities cannot be discerned. |
| DISCUSSION (limitations of evidence) | 23b | • This research has several limitations. Firstly, most studies were reported from Europe and North America, with fewer from other continents. The authors note this his geographic bias affecting the generalisability of results, specifically in resource-limited settings. Generalisability is further affected by the majority of included articles being case reports which are acknowledged as less robust than other study designs. The overall dearth of data has prevented the application of more rigorous statistical methods allowing only for a narrative analysis. |
| DISCUSSION (limitations of review process) | 23c | • Limited only to papers published after 2000 and in English. |
| DISCUSSION (implications) | 24d | • Further research required before implications can be inferred. |
| **OTHER INFORMATION** | | |
| REGISTRATION AND PROTOCOL (registration) | 24a | • The review was not registered. |
| REGISTRATION AND PROTOCOL (protocol) | 24b | • A protocol was not prepared. |
| REGISTRATION AND PROTOCOL (amendments) | 24c | • No amendments were made. |
| SUPPORT | 25 | • Financial support was provided to the authors and project accordingly: SLE is supported by Research Program Training scholarship at the University of Western Australia, a Wesfarmers Centre of Vaccines and Infectious Diseases Top Up scholarship and the Stan and Jean Perron Foundation. SB is supported by a Higher Degree by Research Scholarship at the University of Queensland. JRC and ACB are supported by NHMRC Investigator Awards (GNT1173874 and GNT1175509 respectively). This study was supported by an NHMRC Synergy Grant (GNT2010716). The funders had no role in study design, data collection and analysis, decision to publish, or preparation of the manuscript. |
| COMPETING INTERESTS | 26 | • No competing interests are reported. |
| AVAILABILITY OF DATA, CODE, AND OTHER MATERIALS | 27 | • No other materials are available. |
